# Health effects of a low-inflammatory diet in adults with arthritis: a systematic review and meta-analysis

**DOI:** 10.1017/jns.2020.31

**Published:** 2020-08-27

**Authors:** Furkan Genel, Michael Kale, Natalie Pavlovic, Victoria M. Flood, Justine M. Naylor, Sam Adie

**Affiliations:** 1 St George and Sutherland Clinical School, University of New South Wales, Sydney, NSW 2052, Australia; 2 Whitlam Orthopaedic Research Centre, Orthopaedic Department, Liverpool Hospital, Liverpool, NSW 2170, Australia; 3 Gosford and Wyong Hospital, Central Coast Local Health District, Gosford, NSW 2250, Australia; 4 School of Medicine and Public Health, Faculty of Health and Medicine, University of Newcastle, Callaghan, NSW 2308, Australia; 5 South Western Sydney Clinical School, University of New South Wales, Liverpool, NSW 2170, Australia; 6 Fairfield Hospital, South Western Sydney Local Health District, Prairiewood, NSW 2176, Australia; 7 Sydney School of Health Sciences, Faculty of Medicine and Health, University of Sydney, Sydney, NSW 2006, Australia; 8 Westmead Hospital, Western Sydney Local Health District, Westmead, NSW 2145, Australia

**Keywords:** Low-inflammatory diet, Anti-inflammatory diet, Mediterranean diet, Rheumatoid arthritis, Osteoarthritis

## Abstract

The aim is to systematically assess the health impact of a low-inflammatory diet intervention (full-diet or supplement), compared to usual diet or other dietary interventions, on weight change, inflammatory biomarkers, joint symptoms, and quality of life in adults with osteoarthritis, rheumatoid arthritis or seronegative arthropathy (psoriatic, reactive, ankylosing spondylitis or IBD-related), on outcomes assessed in prospective studies within 6 months of intervention commencement (PROSPERO CRD42019136567). Search of multiple electronic library databases from inception to July 2019, supplemented by grey literature searches, for randomised and prospective trials assessing the above objective. After exclusion of 446 ineligible studies, five randomised and two prospective trials involving 468 participants with either osteoarthritis or rheumatoid arthritis were included. GRADE assessment for all outcomes was very low. Meta-analyses produced the following standardised mean differences (SMD) and 95 % confidence interval (CI) 2–4 months following commencement of the diets favouring the low-inflammatory diet: weight SMD −0⋅45 (CI −0⋅71, −0⋅18); inflammatory biomarkers SMD −2⋅33 (CI −3⋅82, −0⋅84). No significant effects were found for physical function (SMD −0⋅62; CI −1⋅39, 0⋅14), general health (SMD 0⋅89; CI −0⋅39, 2⋅16) and joint pain (SMD −0⋅98; CI −2⋅90, 0⋅93). In most studies, the quality of dietary intervention (dietitian input, use of validated dietary compliance tool) could not be gauged. In conclusion, very low-level evidence suggests that low-inflammatory diets or supplements compared to usual diets are associated with greater weight loss and improvement in inflammatory biomarkers. More high-quality trials are needed to assess the health effects of a low-inflammatory diet more comprehensively and conclusively in arthritic conditions.

## Introduction

Arthritis has a significant global burden^(1–3)^. It includes a wide variety of clinical conditions, including osteoarthritis (OA), rheumatoid arthritis (RA) and seronegative arthropathies (SA) (psoriatic, reactive, ankylosing spondylitis (AS) or inflammatory bowel disease (IBD)-related). The pathologies of these diseases are highly variable, but all include a component of joint inflammation. Patients with high disease activity typically have impaired quality of life and activities of daily living^(4–7)^.

The low- or anti-inflammatory diet (both names are synonymous) is based on principles of the Mediterranean diet. It emphasises on foods high in antioxidants, polyphenols, carotenoids, omega-3 fatty acids (long chain), foods low in the glycaemic index and promotes the utilisation of extra virgin olive oil as the main source of fat^(8)^. Furthermore, the diet advises the reduction or minimisation of refined carbohydrates, fast foods, foods high in trans-fat and saturated fat, alcoholic beverages, sugary beverages and processed meats^(9–11)^. Due to the low-inflammatory effects, it is hypothesised that the diet may be beneficial in providing symptom-relief for patients with arthritis. Consequently, many patient-centred medical websites promote such a diet for patients with arthritis^(12–15)^.

Previous research suggests that obese patients with OA can successfully lose weight and report symptom improvement following a diet-based weight loss intervention^(16–18)^. This is also the case for those who suffer from RA^(19)^ and psoriatic arthritis^(20)^. Furthermore, research suggests that a low-inflammatory diet may alleviate arthritic symptoms^(21,22)^. It is unclear; however, if symptom-relief is related to change in inflammation, change in weight, change in adipose tissue, or the quality or type of dietary intervention.

The overall objective of this review is to assess the health impact of a low-inflammatory diet intervention (full-diet or supplements) compared to usual diet or other dietary interventions. Specifically, the health impact includes the effect on inflammatory biomarkers, joint symptoms, quality of life and weight change in adults who suffer from OA, RA or seronegative arthropathy (psoriatic, reactive, AS or IBD-related) affecting any joint in the body, within the first 6 months since the commencement of the intervention.

## Methods

The protocol was registered on PROSPERO prior to commencement of the article screening (Reg. No. CRD42019136567). The PRISMA guidelines were used to report this systematic review^(23)^.

### Eligibility criteria

The eligibility criteria for included studies are summarised in Table 1. Studies that investigated the effects of a low-inflammatory diet intervention in adults with arthritis were assessed for changes in weight, joint symptoms and inflammatory biomarkers. Interventions included any low-inflammatory dietary intervention compared to diet as usual or other forms of dietary interventions. Prospective studies with their full-text articles published in English were eligible for inclusion.

**Table 1.** Systematic review study eligibility criteria

Eligibility criteria	Inclusion criteria	Exclusion criteria
Population	Adults with diagnosis of either Osteoarthritis Rheumatoid arthritis (seropositive or seronegative) Seronegative arthropathies (psoriatic, reactive, ankylosing spondylitis or IBD-related)	Adolescents (under 18 years of age) Other forms of arthritis
Intervention	Interventions termed as ‘low-inflammatory diet’, ‘anti-inflammatory diet’, ‘Mediterranean diet’ or synonyms of these. The diet can be either caloric or non-caloric restricted Duration of intervention of at least 4 weeks The diet intervention is provided in the outpatient setting	Usual diet Other weight loss/diet programs that do not clearly include a low-inflammatory component
Comparator/Control	Usual diet Any other dietary intervention that does not clearly include a low-inflammatory component (such as low carbohydrate and high protein) Interventions including an exercise component will be included if the exercise component is offered in both intervention and control arms	
Outcomes	Patient-reported or physical function, range of motion and pain. Assessment tools such as Oxford score and visual analogue score are inclusive Any blood inflammatory markers, such as C-reactive protein (CRP) and Interleukin-6 (IL-6) Weight change, such as pre- and post-intervention weight or percentage weight change Timepoints will be recorded up to 6 months following the commencement of the intervention Results with varying timepoint of treatment duration will be grouped as 0–2, 2–4 and 4–6 months	
Study Design	Prospective studies (including randomised trials, non-randomised trials and pre–post studies)	Retrospective studies (including studies retrospectively assessing registry data)

### Information sources

The following electronic databases were searched from inception till 10 July 2019; MEDLINE, EMBASE, Cochrane Database of Systematic Reviews (potentially eligible reviews had their references reviewed for eligible studies), Cochrane Central Register of Controlled Trials (CENTRAL), CINAHL. Reference lists of included studies as well as the grey literature (Proceedings of the American Society of Nutrition and International Congress of Dietetics and a Google Scholar search) were also reviewed.

### Search strategy

The search strategy, using a combination of key and text words, was formulated with the help of a medical librarian. The syntax of the search for each database is found in Supplementary Tables and Figures.

### Selection process

Ascertainment of eligible studies was conducted independently by two reviewers (F.G. and M.K.). The titles and abstracts of articles were screened for eligibility. Once potential studies were identified, both reviewers obtained full-text articles to assess and discuss their eligibility. If there was a disagreement between the two reviewers, a resolution was sought by discussing the study with a third reviewer (J.M.N., S.A. or V.M.F.).

### Data collection process and management

Data extraction from eligible studies and subsequent collation was completed by the two reviewers independently using standardised forms. Any discrepancies in the data were resolved through discussion. Attempts were made to contact the corresponding authors of studies where data were missing. When extracting data from trials, only intention-to-treat (ITT) data was utilised. However, if ITT data were not provided in the article or on request, then per-protocol data were utilised.

### Data items

The following data items were extracted from included articles: author, reference, country, population characteristics (such as age, gender, diagnosis and joints affected), study design, trial size (at baseline and size for analysis for both intervention and control/comparator groups), intervention characteristics such as details of the intervention, intervention duration, quality of intervention (see Section ‘Risk of bias’), details of the control/comparator (e.g. compared to normal diet or other diet programs, size of group and duration of control/comparator), duration of follow-up, outcome details (refer to Section ‘Outcomes and prioritisation’), details needed for assessment for risk of bias and author contact details.

### Outcomes and prioritisation

Continuous outcomes were presented as mean differences (MD), standardised mean differences (SMD), for data reported with different tools, along with standard deviations or 95 % confidence intervals (95 % CIs). When standard deviations were not reported, these were calculated from other available data, such as a 95 % CI, standard error, or a *P* value^(24–27)^. There were three primary outcomes:

1. Weight loss – converted to metric units (km) when necessary. Body mass index (BMI) was also extracted, using change scores whenever possible.
2. Blood inflammatory markers – the main markers of interest in this review were C-reactive protein (CRP, both high sensitivity CRP and standard CRP, measured in mg/ml) as well as Interleukin-6 (IL-6, measured in pmol/l) since these are the most widely reported in the literature^(28)^. Data for other inflammatory markers were also extracted including (but not limited to) IL-1β and TNF-alpha.
3. Joint symptoms – using a validated joint pain/function score for arthritis populations, such as Oxford^(29,30)^, or WOMAC scores^(31)^.

Interventional trials had their outcome data extracted at baseline, during the dietary intervention, and at the end of dietary intervention (before or at 6 months). Outcomes reported with varying timepoints were grouped as 0–2, 2–4 and 4–6 months. Scoping of the literature indicated most dietary interventions were for 3 months^(19,21)^; however, interventions existed for 0–3^(32)^ and 3–6 months^(33–35)^. In light of this, and the associated clinical significance of the timepoint, 2–4 months were chosen as the primary timepoint for all outcomes.

### Risk of bias in individual studies

The randomised trials were assessed for bias using the Cochrane Handbook's RoB Version 2 checklist^(36)^, while prospective studies were assessed using the Cochrane Handbook's ROBINS-I tool^(37)^. Each assessment was conducted independently by two reviewers. Any discrepancy was resolved over the discussion and an arbitrator was used if necessary.

As an indicator of therapeutic validity, the quality of the intervention utilised in the studies was also assessed. The areas of interest were informed by an earlier review^(38)^ and included whether (i) a dietitian was involved in the design of the dietary intervention; (ii) the dietary intervention was a partial or full simulation of the low-inflammatory diet; (iii) there was the monitoring of adherence to the dietary intervention and (iv) a validated tool was utilised to measure dietary adherence.

### Data synthesis

Outcomes were pooled from at least two studies in random-effects meta-analysis. The inverse variance method was utilised when conducting a meta-analysis of data from different studies. For the purposes of meta-analysis, timepoints for outcomes were stratified at 0–2, 2–4 and 4–6 months. Studies were pooled when there was satisfactory clinical homogeneity (similar study design, population and intervention); otherwise, they were discussed narratively. Statistical heterogeneity was assessed using the *I*^2^ statistic.

Changes in inflammatory biomarkers and weight were derived by subtracting mean baseline values from subsequent values; thus, a negative change implied improvement in health. However, for joint symptoms, a decrease in score could be an improved outcome (such as WOMAC^(31)^), while an increased score in another tool could also represent improvement (such as Oxford Knee Score^(30)^). Thus, tools that reported poor health in the opposite direction to the majority of other tools had their change scores multiplied by −1^(39)^ (Supplementary Table S1).

Some data in the selected studies were not presented in a usable form without modification. For example, change in weight (as a percentage) was reported instead of pre- and post-weight. To ensure the same variables were extracted from all studies, these variables were converted to a usable form (e.g. the post-weight was calculated from the reported basal weight and percentage change in weight). When this could not be done or missing data was encountered, missing data were requested from corresponding authors. No imputation of missing data was undertaken. Data analysis was completed using RevMan 5.3 software^(40)^.

*A priori*, we planned to explore heterogeneity by performing a subgroup analysis of (i) osteoarthritis *v.* inflammatory/seronegative arthritis, (ii) different duration of intervention (0–2 months *v*. 2–4 months (timepoint of interest) *v*. 4–6 months) to assess possible dose-response effects and (iii) RCTs *v*. other prospective studies. After studies were identified, we also planned subgroup analysis of (i) diets that resulted in weight loss *v*. diets without weight loss and (ii) full low-inflammatory diet *v.* partial diet (e.g. anti-inflammatory supplements).

### Meta-biases and confidence in cumulative evidence

Assessment of publication bias was planned, but there were insufficient studies (<10 studies), to construct a funnel plot or perform Egger's regression test. The methodology of the Grading of Recommendations Assessment, Development and Evaluation (GRADE)^(41)^ working group was utilised in assessing the quality of the evidence presented in the review. The evidence was graded as either very low, low, moderate or high-quality evidence.

## Results

### Study selection

**Fig. 1.**
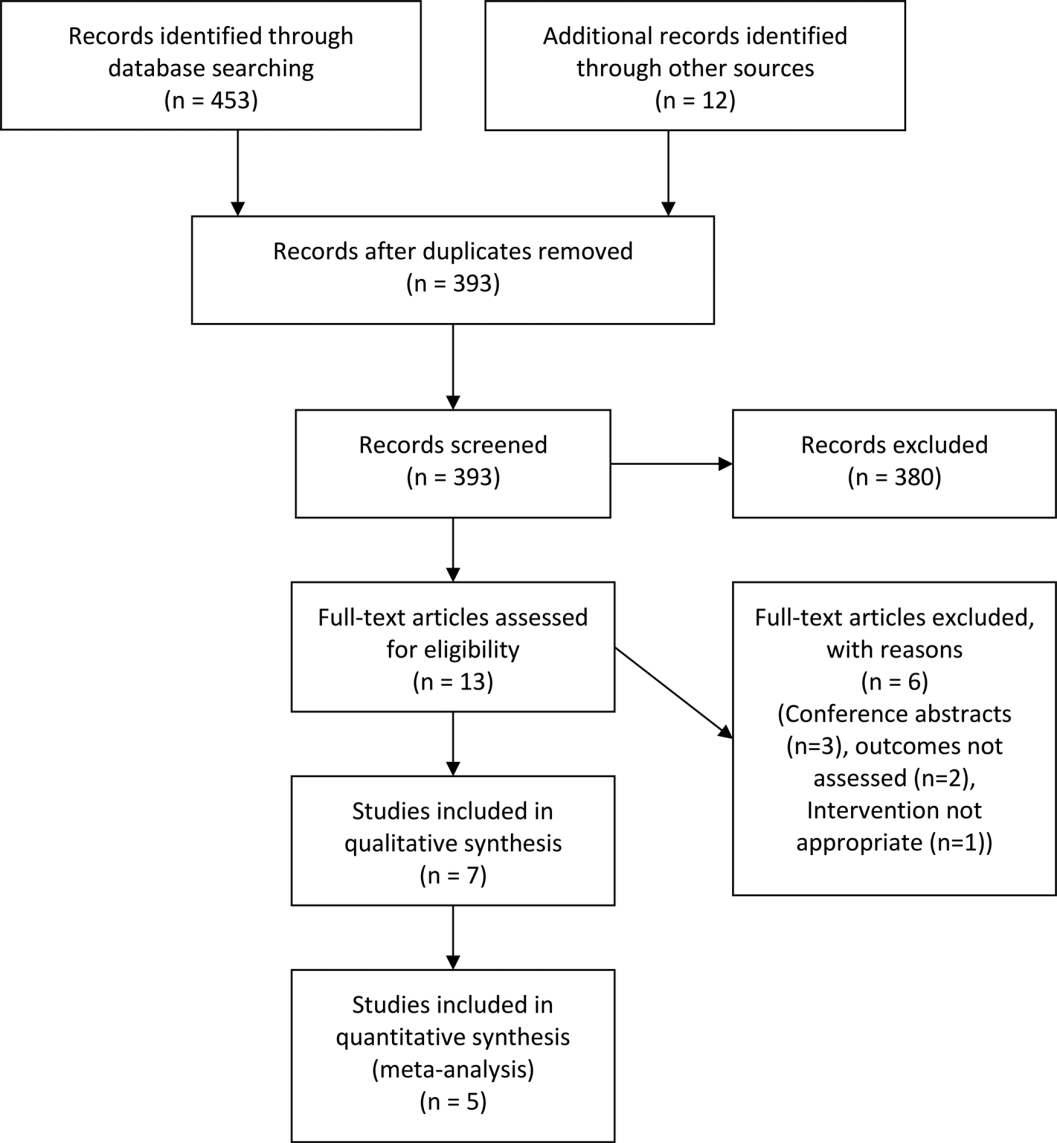
PRISMA flow diagram of the article screening and selection process for the systematic review.

Of the 453 citations retrieved from multiple sources, seven articles^(19,21,22,33,42–44)^ met the eligibility criteria (Fig. 1). Five articles were randomised trials^(19,22,33,42,44)^, two were pre–post trials^(21,43)^. These trials had a total of 468 patients (259 in the intervention and 226 in the control). Requests for additional information were made to four authors; one responded.

### Study characteristics

The age range for the participants was from 30 to 90 years. Five of the seven trials included both sexes; two studies included women only. ‘Women only’ studies focused on RA, while the other trials focused either on RA or OA. Intervention duration ranged from 3 to 6 months. Five of the seven studies had a low-inflammatory diet (including ‘anti-inflammatory’ and ‘Mediterranean’ diet) as the intervention, while two studies utilised low-inflammatory supplements (i.e. strawberry and blueberry powders). All intervention diets were non-calorie restricted. Control/comparator diets included the usual diet or placebo powders. There were a total of 259 and 226 participants in the intervention and control/comparator analyses, respectively. The characteristics of the included trials are found in Table 2. One of the studies^(44)^ was a cross-over trial. As per Cochrane guidelines, the data were extracted as if the study was a parallel-group trial of intervention *v.* control^(45)^.

**Table 2.** Study characteristics of included studies of the systematic review

Author and Year	Adam *et al*., 2003	Du *et al*., 2019	Dyer *et al*., 2017	Garcia Morales *et al*., 2019	McKellar *et al*., 2007	Schell *et al*., 2017	Skoldstam *et al*., 2003
Country	Germany	USA	England, UK	Mexico	Glasgow, UK	USA	Sweden
Study Design	Prospective Trial (Case-matched Prospective Study (diet component) with randomised double-blind cross-over study (fish oil component))	RCT (Double-Blind Randomised Control Group Pre-test Post-test Design)	RCT	RCT	Prospective Trial (Non-randomised Control Group Pre-test Post-test Design)	RCT (Randomised, Double-Blind Cross-over Trial)	RCT
Final *N* of Participants	68 recruited, 60 completed	63 recruited, 49 completed	124 recruited, 99 completed	71 recruited and completed	130 recruited and completed	17 recruited and completed	56 recruited, 51 completed
Gender	M + F	M + F	M + F	F	F	M + F	M + F
Age Group	57 ± 12⋅8	45–79	31–90	>18	30–70	57 ± 7	33–75
Diagnosis	Rheumatoid Arthritis	Osteoarthritis	Osteoarthritis	Rheumatoid Arthritis	Rheumatoid Arthritis	Osteoarthritis	Rheumatoid Arthritis
Joints affected	Unspecified	Knee	Unspecified	Unspecified	Unspecified	Knee	Unspecified
Intervention/Control Duration	3 months	4 months	4 months	6 months	6 months	3 months	3 months
Intervention Diet type	Low-Inflammatory Diet (LID)	Blueberry Powder (AID Food)	Mediterranean Diet (MD)	Mediterranean Diet (group 3 of the trial)	Mediterranean-type Diet	Strawberry Powder (AID Food)	Mediterranean Diet
Details of Nature of Intervention	LID ± Fish oil	One 20 g freeze-dried blueberry powder twice a day made into drink	Diet information provided, dietitian phone support	Individualised MD diet, menus, scheduled visits	2 h weekly cooking class for 6 weeks	12 weeks of blinded strawberry concentrate	3-week intense outpatient diet education, dietitian weekly phone calls and 3rd weekly appointments
Control/Comparator Diet type	Regular Diet + Placebo	Placebo powder	Regular diet	Regular diet	Healthy Diet (given written information)	Placebo powder	Regular diet
Details of Control/Comparator type	Regular Diet + Placebo oil tablet	1 × 20 g Placebo powder twice a day	Regular diet	General advice	Written information provided	Control powder composed like that of intervention powder in regard to texture and taste but without strawberry in the powder	Regular diet
Intervention trial size at baseline	34	33	Information not provided	40	75	17	29
Intervention trial size at analysis	30	27	49 (Biomarkers had 29 and BMI had 22)	35	75	17	26
Control trial size at baseline	34	30	Information not provided	31	55	17	27
Control trial size at analysis?	30	22	50 (Biomarkers had 25 and BMI had 17)	27	55	17	25
Outcomes from study utilised in meta-analysis	Weight	Weight	Weight, Inflammatory Biomarkers, Physical Function, Pain	Nil	Nil	Weight, Inflammatory Biomarkers, Physical Function, General Health, Pain	Weight, Inflammatory Biomarkers, Physical Function, General Health, Pain

RCT, Randomised Control Trial; M, Male; F, Female; LID, Low-inflammatory Diet; AID, Anti-inflammatory Diet; MD, Mediterranean Diet; BMI, Body Mass Index.

### Risk of bias

From the randomised trials, one study^(44)^ had an overall low risk of bias, while the remaining studies^(19,22,33,42)^ had an overall high risk of bias (Supplementary Fig. S1). For the prospective non-randomised trials, one^(21)^ had an overall moderate risk of bias, while the other^(43)^ had an overall serious risk of bias (Supplementary Table S2).

In assessing the quality of the dietary interventions specifically, only one study^(33)^ clearly stated the utilisation of a dietitian in designing the intervention. Two studies^(22,33)^ were a full simulation of a low-inflammatory diet, while other studies were only partial. While all the studies that assessed participant dietary compliance did so in various ways, only one study^(43)^ utilised a validated tool (Supplementary Table S3).

### Results of individual studies

Individual study level data is provided in Supplementary Tables S4 and S5. Synthesised results are reported below.

### Synthesis of results

Table 3 presents the *GRADE Summary of Findings* for each outcome. SMD values were converted back to familiar clinical measurements^(24,25)^. Overall, the outcome results were of very low quality as assessed by the GRADE system. There were a small number of studies included in the analysis, and high heterogeneity was evident for most comparisons.

**Table 3.** GRADE Summary of Findings

Outcome measure	Number and design of trials	No. of participants	Time of Intervention	Inconsistency	Indirectness	Imprecision	Industry funding	SMD (95 % CI)	Familiar clinical measurements	Interpretation of represented SMD	GRADE Quality of Evidence	Importance
Weight	4 Randomised Trials^(19,22,42,44)^ 1 Prospective Study^(21)^	231	2–4 months	No	Yes	Yes	Yes	−0⋅45 [−0⋅71, −0⋅18]	kg: −2⋅79 kg [−4⋅41, −1⋅12]	The low-inflammatory diet's mean weight was on average 2⋅79 kg lower	Very Low 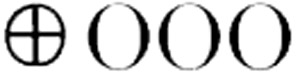	Important but not critical. 5–10 % weight loss is considered significant clinically^(46,47)^ (e.g. 3⋅5 kg in a 70 kg person)
Inflammatory Biomarkers	3 Randomised Trials^(19,22,44)^	136	2–4 months	Yes	No	Yes	No	−2⋅33 [−3⋅82, −0⋅84]	CRP: −50⋅65 mg/l [−83⋅04, −18⋅26]	The low-inflammatory diet's mean CRP level was on average 50⋅65 mg/l lower	Very Low 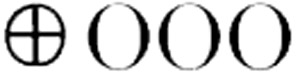	Important but not critical. MCID not known in this context
Physical Function	3 Randomised Trials^(19,22,44)^	183	2–4 months	Yes	No	Yes	No	−0⋅62 [−1⋅39, 0⋅14]	HAQ: −0⋅29 [−0⋅65, 0⋅07]	The low-inflammatory diet's mean HAQ score was on average 0⋅29 lower	Very Low 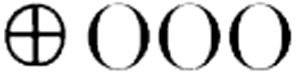	Important but not critical. MCID as 0⋅22^(48,49)^
General Health	2 Randomised Trials^(19,22)^	85	2–4 months	Yes	No	Yes	No	0⋅89 [−0⋅39, 2⋅16	VAS Health: 0⋅24 mm [−0⋅11, 0⋅59]	The low-inflammatory diet's mean VAS Health score was on average 0⋅24 mm higher	Very Low 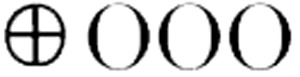	Important but not critical. MCID not known in this context
Pain	3 Randomised Trials^(19,22,44)^	183	2–4 months	Yes	No	Yes	No	−0⋅98 [−2⋅90, 0⋅93]	Pain VAS: −66⋅86 mm [−197⋅87, 63⋅45]	The low-inflammatory diet's mean Pain VAS score was on average 66⋅86 mm lower	Very Low 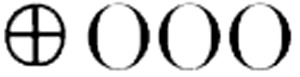	Important but not critical. MCID as 11 mm for patients with RA^(50)^

Outcomes utilised were data from 2 to 4 months of intervention. The ‘GRADE Quality of Evidence’ was derived based on the GRADE methodology. (Interpretation of SMD results were calculated by multiplying the SMD with sd of the tool its being re-expressed as. sd was derived from the pooled mean and 95 % CI of all study groups (intervention and control groups) for that specific tool.)

#### Weight

**Fig. 2.**
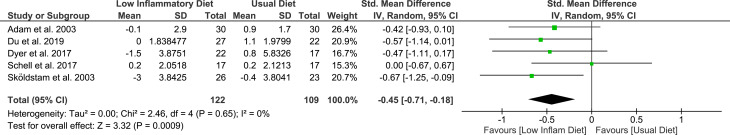
Meta-analysis of weight change when comparing low-inflammatory diet to usual diet following 2–4 months of intervention/control.

A standardised analysis combining weight (kg) and BMI across the studies after 2–4 months of the intervention, showed very low-quality evidence favouring weight change in the low-inflammatory group, with a statistically significant SMD of −0⋅45 ([95 % CI −0⋅71, −0⋅18], *P* = 0⋅0009) compared to the usual diet group (Fig. 2).

A subgroup analysis assessing weight change according to diagnosis indicated patients diagnosed with RA and OA had a weight change following a low-inflammatory diet (RA – SMD −0⋅53 [95 % CI −0⋅91, −0⋅14], *P* = 0⋅007; OA – SMD −0⋅37 [95 % CI −0⋅73, −0⋅01], *P* = 0⋅04; Supplementary Fig. S2), although there were no differences between subgroups (test for subgroup difference, *P* = 0⋅57). When analysing based on study type, only randomised trials favoured the low-inflammatory diet (RCTs – SMD −0⋅46 [95 % CI −0⋅76, −0⋅15], *P* = 0⋅004; prospective trial – SMD −0⋅42 [95 % CI −0⋅93, 0⋅10], *P* = 0⋅11; Supplementary Fig. S3). Further subgroup analysis based on the nature of intervention (full *v*. partial low-inflammatory diet) can be found in Supplementary Fig. S9. Weight change at 2–4 months favoured full simulation of low-inflammatory diet, but not partial simulation (full simulation SMD −0⋅51 [95 % CI −0⋅84, −0⋅18], *P* = 0⋅002; partial simulation SMD –0⋅31 [95 % CI −0⋅87, 0⋅24], *P* = 0⋅27; Supplementary Fig. S9).

Only one study^(33)^ reported data for weight change after 4–6 months of intervention. The calculated change scores can be found in Supplementary Tables S4 and S5.

#### Inflammatory biomarkers

**Fig. 3.**
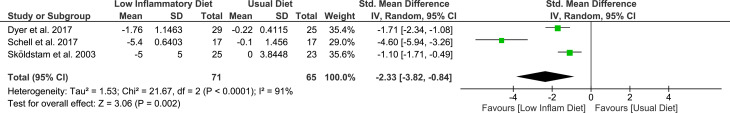
Meta-analysis of inflammatory biomarker change when comparing low-inflammatory diet to usual diet following 2–4 months of the intervention/control. In this analysis of change scores, Skoldstam's CRP results, Schell's IL-6 and Dyer's IL-6 results were utilised.

There was very low-quality evidence that reduction in inflammatory biomarkers at 2–4 months favoured the low-inflammatory diet; SMD −2⋅33 ([95 % CI −3⋅82, −0⋅84], *P* = 0⋅002; Fig. 3). An individual meta-analysis for each type of inflammatory biomarker did not favour either diet. At 2–4 months, the low-inflammatory group had an MD in change scores of −2⋅46 mg/l ([95 % CI −7⋅15, 2⋅24], *P* = 0⋅31), −3⋅41 pg/ml ([95 % CI −7⋅09, 0⋅28], *P* = 0⋅07) and –4⋅56 pg/ml ([95 % CI −12⋅43, 3⋅32], *P* = 0⋅26) in CRP, IL-6 and IL-1β, respectively (Supplementary Fig. S4).

In a subgroup analysis based on diagnosis, both the OA group (SMD −3⋅09 [95 % CI −5⋅92, −0⋅26], *P* = 0⋅03) and the RA group (SMD −1⋅10 [95 % CI −1⋅71, −0⋅49], *P* = 0⋅0004; Supplementary Fig. S5) had reductions in inflammatory biomarkers (independent of weight change); the differences between the group were not significant (*P* = 0⋅18; Supplementary Fig. S5). Additional subgroup analyses (based on the nature of intervention and weight loss during intervention) can be found in Supplementary Fig. S10. Inflammatory biomarkers at 2–4 months favoured both partial and full simulation of low-inflammatory diet (partial simulation SMD −4⋅60 [95 % CI −5⋅94, −3⋅26], *P* < 0⋅00001; full simulation SMD −1⋅40 [95 % CI −2⋅00, −0⋅80], *P* < 0⋅00001; Supplementary Fig. S10). Change in biomarkers was favoured in both the presence and absence of weight loss in the low-inflammatory diet (Supplementary Fig. S10).

Only one study^(19)^ reported inflammatory marker change scores after 0–2 months for intervention. The calculated change scores can be found in Supplementary Tables S4 and S5.

#### Patient-reported outcome measures

**Fig. 4.**
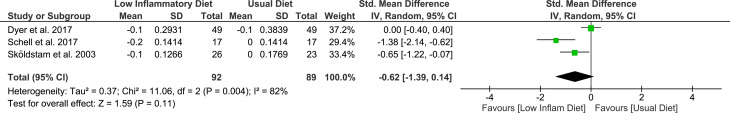
Meta-analysis of physical function change when comparing low-inflammatory diet to usual diet following 2–4 months of the intervention/control.

At 2–4 months, very low-quality evidence suggests that physical outcome measures did not favour either diet (SMD −0⋅62 [95 % CI −1⋅39, 0⋅14]), *P* = 0⋅11; Fig. 4). This analysis was completed utilising the intervention and control change scores of Arthritis Impact Measurement Scales (AIMS-2) physical score from Dyer *et al*. (2017; intervention mean −0⋅1, standard deviation (sd) 0⋅2931; control mean −0⋅1, sd 0⋅3839^(22)^) and Health Assessment Questionnaire (HAQ) scores from Schell *et al*. (2017; intervention mean −0⋅2, sd 0⋅1414; control mean 0, sd 0⋅1414^(44)^) and Skoldstam *et al*. (2003; intervention mean −0⋅1, sd 0⋅1266; control mean 0, sd 0⋅1769^(19)^; Supplementary Tables S4 and S5). Subgroup analysis by diagnosis demonstrates inconclusive results for OA (SMD −0⋅65 [95 % CI −2⋅00, 0⋅70], *P* = 0⋅34). However, in RA, physical outcome measures favoured the low-inflammatory diet (SMD −0⋅65 [95 % CI −1⋅22, −0⋅07], *P* = 0⋅03; Supplement Fig. S6). The test for subgroup differences produced a *P* value of 0⋅99, suggesting that the results did not favour either the OA or RA group (Supplementary Fig. S6). Additional subgroup analysis based on the nature of intervention (full *v.* partial low-inflammatory diet) and the presence/absence of weight loss in the intervention can be found in Supplementary Fig. S1^1^. Physical function at 2–4 months favoured the partial simulation of low-inflammatory diet and not the full simulation (partial simulation SMD −1⋅38 [95 % CI −2⋅14, −0⋅62], *P* = 0⋅0004; full simulation SMD −0⋅29 [95 % CI −0⋅92, 0⋅34], *P* = 0⋅37). Furthermore, change in physical function was favoured in the absence of weight loss in the low-inflammatory diet and not in the presence of weight loss in the low-inflammatory diet.

**Fig. 5.**
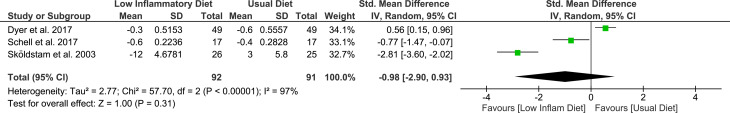
Meta-analysis of pain score change when comparing low-inflammatory diet to usual diet following 2–4 months of the intervention/control.

A meta-analysis was also completed assessing change in pain scores. At 2–4 months, there was very low-quality evidence that change in any pain score favoured neither diet (SMD −0⋅98 [95 % CI −2⋅90, 0⋅93], *P* = 0⋅31; Fig. 5). In a subgroup analysis based on diagnosis, pooled pain scores were different; the RA group had a statistically significant SMD of −2⋅81 ([95 % CI −3⋅60, −2⋅02], *P* < 0⋅00001; Supplementary Fig. S8) favouring the low-inflammatory diet. Subgroup difference analysis resulted in a *P* value = 0⋅0004, suggesting that the results favoured the RA group over the OA group. Furthermore, subgroup analysis based on the nature of intervention (full *v*. partial low-inflammatory diet) and the presence/absence of weight loss in the intervention can be found in Supplementary Fig. S1^2^. Pain scores at 2–4 months favoured only the partial simulation and not the full simulation of low-inflammatory diet (partial simulation SMD −0⋅77 [95 % CI −1⋅47, −0⋅07], *P* = 0⋅03; full simulation SMD −1⋅11 [95 % CI −4⋅41, 2⋅19], *P* = 0⋅51). Additionally, change in pain scores was favoured in both the presence and absence of weight loss in the low-inflammatory diet.

At 2–4 months, there was very low-quality evidence that general health outcomes favoured neither the diet group (SMD 0⋅89 [95 % CI −0⋅39, 2⋅16]), *P* = 0⋅17; Supplementary Fig. S7). Results for 0–2 months of intervention were calculated for physical function and pain scores (Supplementary Figs. S13 and S14); physical function and pain scores favoured neither the diet group. One study^(33)^ reported change scores in weight change, physical outcome, pain and general health at 4–6 months of intervention. It showed that the health benefits for the low-inflammatory diet may be present at 4–6 months of intervention; however, a meta-analysis to assess this could not be conducted due to insufficient studies. Calculated change scores can be found in Supplementary Tables S4 and S5.

## Discussion

This systematic review provides a comprehensive assessment of the dearth of literature concerning the health effects of a low-inflammatory diet in people with arthritis. In comparison to a usual diet, very low-quality GRADE evidence suggests that a low-inflammatory diet is associated with more weight loss, lower inflammation, improved physical function measures (RA only) and reduced joint pain (RA only). We also assessed the effect sizes at different periods and found that changes in outcomes were found as early as 2–4 months following commencement of the intervention.

Obesity is common among those who suffer from OA^(51)^ and RA^(52,53)^. In the meta-analysis, one study^(44)^ specifically recruited obese patients with knee OA, while another study's baseline characteristics indicate the inclusion of obese participants^(42)^. Previous research indicates calorie-restricted diets are effective in producing weight loss in OA patients^(18,54)^. A recent network meta-analysis suggests that low-inflammatory diets, though often not targeting weight loss, may also usefully reduce weight within 6 months of commencement though the effects diminish by 12 months^(55)^. We also found in this meta-analysis that the low-inflammatory diet could procure weight loss. This is important because such a diet can be useful for not only reducing the inflammatory load induced by diet but also the inflammatory load associated with excess adipose tissue^(55)^.

Inflammatory biomarkers are elevated in the serum of patients with arthritis. IL-6 and CRP have roles in the pathogenesis of arthritis and are associated with disease activity^(56,57)^. Reduction in inflammatory biomarkers may indicate reduced disease severity and progression. Previous research indicates that a calorie-restricted diet and exercise reduces IL-6 levels in obese OA patients^(18)^. One clinical trial highlighted the reduction of IL-6 and hsCRP following significant weight loss secondary to gastric surgery^(58)^. While improvements in inflammatory biomarkers may be confounded by the concomitant weight loss, our results reveal improvements in biomarkers can occur independent of weight change.

For RA patients, improvement in pain and physical function measures were greater with those following a low-inflammatory diet. Statistically significant benefits were not seen in people with OA. Our findings are not consistent with studies where people with OA were given low or calorie-restricted diets and significant improvements in pain and function were observed^(18,54,59)^. These contradictory findings may suggest that for OA patients to obtain physical function and pain benefits from a dietary intervention, calorie restriction needs to be incorporated. Interestingly, general health measures were not associated with either the diet group; however, only two papers were part of the meta-analysis.

Varied duration of the interventions was used to assess the potential dose-response effect. As stated in the results, change scores were extracted for three time periods; however, the majority of the data were applied to the 2–4 months period. Comparing the meta-analysis results from 0–2 months and 2–4 months, the data suggest that change in outcomes were evident after 2 months of intervention. Due to lack of data beyond 4 months, we could not determine what the ideal duration of intervention is to produce the most significant improvements in outcomes.

Most of the dietary interventions only provided a partial simulation of the low-inflammatory diet and did not involve a dietitian in its design; hence, the therapeutic validity of these dietary interventions is questionable. Treatment fidelity is also questionable as there was a lack of a validated tool used to assess dietary compliance during the trials. Strengths of this review are that it covers a topic of great public interest given the interest in diet and arthritis generally^(12,13)^ and includes a pre-registered protocol which was performed and reported within current guidelines (including PRISMA guidelines and Cochrane Handbook). Furthermore, change scores were imputed from the varied duration of intervention/control (to assess potential dose-effect responses), and we dealt with heterogeneity where possible using sensitivity analyses. Furthermore, strengths include the *a priori* design which was pre-registered, the comprehensive assessment of the quality of the evidence and incorporation of GRADE, and the standardisation of outcomes in order to include them in pooled effect sizes – which were then re-converted into meaningful clinical measures. Limitations of the review are that the included studies had small samples and were predominantly of poor quality, which resulted in imprecision given the small number of participants (less than 400), a heterogeneous analysis and missing data – all which limit the confidence in our overall conclusions.

## Conclusion

In this meta-analysis of randomised and prospective trials, there is the very low quality of evidence, suggesting that a low-inflammatory diet is associated with greater weight loss, a greater decrease in inflammatory biomarkers, greater improvement in joint pain measures (RA only) and greater improvement in physical function measures (RA only) compared to usual diets. Health benefits appear to favour those with rheumatoid arthritis more than osteoarthritis. Although many patient-oriented information sources promote the low-inflammatory diet for arthritis patients, this review highlights the poor quality of evidence behind these public health recommendations. Furthermore, high-quality trials are needed evaluating a dietitian-led, low-inflammatory diet on a combination of laboratory and patient-reported outcomes, particularly in osteoarthritis.

## Supplementary material

For supplementary material accompanying this paper visit http://dx.doi.org/10.1017/jns.2020.31. click here to view supplementary material
