# Supplementary material for: Health effects of a low-inflammatory diet in adults with arthritis: a systematic review and meta-analysis
Source: J Nutr Sci. 2020 Aug 27;9:e37. doi: 10.1017/jns.2020.31 (PMC7503186; doi:10.1017/jns.2020.31)
Supplement: Supplementary file 1 [file S2048679020000312sup001.pdf]

The health effects of a low-inflammatory diet in adults with arthritis: systematic review and meta-analysis

Furkan Genel, Michael Kale, Natalie Pavlovic, Victoria M Flood, Justine M Naylor, Sam Adie

Supplementary Tables, Figures and Search Syntax:

Supplementary Table 1: Table illustrating which measures were multiplied by -1

PROM	Low Score	High score	Multiply by -1?
**Physical Health:**			
**- HAQ**		Poor Health	No
**- AIMS2 Physical**		Poor Health	No
**- SF-36 Physical**	Poor Health		Yes
**General Health:**			
**- VAS Global**	Poor Health		No
**- VAS Health**	Poor Health		No
**- SF-36 Global Health**	Poor Health		No
**Pain Scores:**			
**- SF-36 bodily pain**	Poor Health		Yes
**- Pain VAS**		Poor Health	No
**- AIMS2 Symptom**		Poor Health	No
**- ICOAP Total**		Poor Health	No

Supplementary Table 2: Risk of Bias assessment for non-randomised trials utilising Cochrane's ROBINS-I tool

**Domain**	**Adam et. al. 2003**	**McKellar et. al. 2007**
Bias due to confounding	Low risk	Serious risk
Bias in selection of participants into the study	Low risk	Low risk
Bias in classification of interventions	Low risk	Moderate risk
Bias due to deviations from intended interventions	Moderate risk	Low risk
Bias due to missing data	Moderate risk	Low risk
Bias in measurement of outcomes	Moderate risk	Moderate risk
Bias in selection of the reported result	Moderate risk	Low risk
Overall	*Moderate risk*	*Serious risk*

Supplementary Table 3: Assessing risk of bias in the dietary interventions implemented in the studies.

**Author and Year**	**Was a dietitian involved in the designing of the dietary intervention ?**	**Was the dietary intervention a partial or full simulation of the low inflammatory/Mediterranean diet?**	**Was there monitoring of adherence to the dietary intervention?**	**Was a validated tool utilised to measure dietary adherence?**
Dyer et. al., 2017	Not Specified	Full simulation of Mediterranean Diet	Yes, a 7 day food diary (start, middle and end) + compliance score	No - self developed compliance score 0-100
Skoldstam et. al., 2003	Not Specified	Modified Mediterranean for Swedish people.	Yes, 3 week BD meal program, weekly dietician phone and 3rd weelky visits	No, had questionairres and dietary interviews to assess adherence, but no specific mention of any validated tool utilised.
Garcia Morales et. al., 2019	Yes	Full simulation of Mediterranean Diet	Yes, however used a 24hr food recall questionairre	No
Schell et. al., 2017	Not Specified	Partial simulation of Low inflammatory diet	Yes - 3 day food dirary (week 6, 12, 14, 20, 26)	No
Du et. al., 2019	Not Specified	Partial simulation of Low inflammatory diet	Yes, calenders were given to patients to remind them of consumption, but compliance was based on self reporting.	No
Mckellar et. al., 2007	Not Specified	Partial simulation of Mediterranean Diet (refer to it as "Mediterranean-type diet")	Yes, using FFQ questionairres	Yes
Adam et. al., 2003	Not Specified	Modified AID (lactoveg)	Yes - monthly conseling with 3 days recorded prior to vist	No

Supplementary Table 4: Individual study intervention results

Outcome	Specific Measure	Study	N=	0-2 Months MEA	0-2 Months SD	2-4 Months MEA	2-4 Months S	4-6 months MEA	4-6 months S
Weight Change	Kg	Adam 2003	30			-0.1	2.9		
		Garcia Morales 2019	35					-2.4	2.4265
		Skoldstam 2003	26			-3	3.8425		
		Dyer 2017	22			-1.5	3.8751		
Weight Change	BMI	Du 2019	27			0	1.838477		
		Schell 2017	17			0.2	2.0518		
Change in Inflammatory Markers	ESR	Skoldstam 2003	26	7	5.3852	1	4.1602		
	(hs/)CRP	Skoldstam 2003	24 (0-2m) / 25 (2-4m)	10	11.9181	-5	5		
		Schell 2017	17			-1.1	1.5		
	IL-6	Schell 2017	17			-5.4	0.6403		
		Dyer 2017	29			-1.76	1.1463		
	IL-1B	Schell 2017	17			-11.1	4.0608		
		Dyer 2017	29			-0.47	0.3666		
PROM	HAQ(/-DI) - (measure functional status in RA, OA, etc.)	Skoldstam 2003	26 (n=25 for 0-2m)	-0.1	0.1401	-0.1	0.1266		
		Garcia Morales 2019	35					0.55	0.6
		Schell 2017	17			-0.2	0.1414		
	AIMS2 Physical (subscore assessing physical function - high score equals poor health - can correlate with HAQ scores)	Dyer 2017	49	0	0.3239	-0.1	0.2931		
	SF-36 physical function	Skoldstam 2003	26			-2.5	15.2		
		Garcia Morales 2019	35					-4.6	22.8
	DAS28 (Measure of RA disease activity)	Skoldstam 2003	26	-0.2	0.3616	-0.5	0.3328		
	VAS Global	Skoldstam 2003	26	-4	5.9646	-12	5.0115		
	VAS Health	Schell 2017	17			0.3	0.2236		
	SF-36 Global Health	Skoldstam 2003	26			5.7	14.6		
		Garcia Morales 2019	35					-0.5	25.7
	SF-36 Bodily Pain	Skoldstam 2003	26			-4.5	24.3		
		Garcia Morales 2019	35					-4.2	24.3
	Pain VAS	Skoldstam 2003	26	-2	5.831	-12	4.6781		
		Schell 2017	17			-0.6	0.2236		
	AIMS2 Symptom (Pain)	Dyer 2017	49	-0.1	0.5459	-0.3	0.5153		
	ICOAP (Total Pain)	Schell 2017	17			-16	4.827		
ROM	Knee Flexion	Dyer 2017	33			10	5.2223		
	Hip Rotation	Dyer 2017	33			14	4.324		
	Hip Flexion	Dyer 2017	33			5	3.8925		

Supplementary table 5: Individual study control/usual diet results

Outcome	Specific Measures	Study	N=	0-2 Months MEAN	0-2 Months SD	2-4 Months MEAN	2-4 Months SD	4-6 months MEAN	4-6 months SD
Weight Change	Kg	Adam 2003	30			0.9	1.7		
		Garcia Morales 2019	27					-0.8	2.4591
		Skoldstam 2003	23			-0.4	3.8041		
		Dyer 2017	17			0.8	5.8326		
Weight Change	BMI	Du 2019	22			1.1	1.9799		
		Schell 2017	17			0.2	2.1213		
Change in Inflammatory Markers	ESR	Skoldstam 2003	25	-1	4.2426	2	4.8415		
	(hs/)CRP	Skoldstam 2003	23	-3	3.4704	0	3.8448		
		Schell 2017	17			-0.9	1.4422		
	IL-6	Schell 2017	17			-0.1	1.456		
		Dyer 2017	25			-0.22	0.4115		
	IL-1B	Schell 2017	17			-2.4	4.1761		
		Dyer 2017	25			0.19	0.4136		
PROM	HAQ(/-DI) - (measure functional status in RA, OA, etc.)	Skoldstam 2003	23	-0.1	0.1769	0	0.1769		
		Garcia Morales 2019	27					0.82	0.68
		Schell 2017	17			0	0.1414		
	AIMS2 Physical (subscore assessing physical function - high score equals poor health - can correlate with HAQ scores)	Dyer 2017	49	-0.1	0.3839	-0.1	0.3839		
	SF-36 physical function	Skoldstam 2003	25			-1.4	13.4		
		Garcia Morales 2019	27					0	17.3
	DAS28 (Measure of RA disease activity)	Skoldstam 2003	23	-0.1	0.4128	0	0.4278		
	VAS Global	Skoldstam 2003	25	-2	5.8	-1	5.8		
	VAS Health	Schell 2017	17			0	0.1414		
	SF-36 Global Health	Skoldstam 2003	25			0.7	21.7		
		Garcia Morales 2019	27					-7.8	18.8
	SF-36 Bodily Pain	Skoldstam 2003	25			-4	20.1		
		Garcia Morales 2019	27					4.4	30.8
	Pain VAS	Skoldstam 2003	25	2	6.2482	3	5.8		
		Schell 2017	17			-0.4	0.2828		
	AIMS2 Symptom (Pain	Dyer 2017	49	-0.6	0.5459	-0.6	0.5557		
	ICOAP (Total Pain)	Schell 2017	17			-5.5	4.3139		
ROM	Knee Flexion	Dyer 2017	28			-5	6.2593		
	Hip Rotation	Dyer 2017	28			-1	5.3486		
	Hip Flexion	Dyer 2017	28			1	2.8094		

Studies with intention-to-treat	Unique ID	Randomization process	Deviations from intended interve	Missing outcome data	Measurement of the outcome	Selection of the reported result	Overall
	Dyer et. al. 2017	?	−	−	?	−	−
	Skoldstam et. al. 2003	?	+	−	+	?	−
	Garcia Morales et. al. 2019	+	+	−	+	−	−
	Schell et. al. 2017	+	+	+	+	+	+
	Du et. al. 2019	?	−	−	+	−	−

+ Low risk
? Some concerns
− High risk

Supplementary Figure 1: Risk of Bias assessment for randomised trials using Cochrane's ROB-V2 methodology

Study or Subgroup	Low Inflammatory Diet Mean	SD	Total	Usual Diet Mean	SD	Total	Weight	Std. Mean Difference IV, Random, 95% CI
**1.4.1 Osteoarthritis**								
Du et al. 2019	0	1.838477	27	1.1	1.9799	22	20.9%	-0.57 [-1.14, 0.01]
Dyer et al. 2017	-1.5	3.8751	22	0.8	5.8326	17	16.7%	-0.47 [-1.11, 0.17]
Schell et al. 2017	0.2	2.0518	17	0.2	2.1213	17	15.3%	0.00 [-0.67, 0.67]
**Subtotal (95% CI)**			**66**			**56**	**52.9%**	**-0.37 [-0.73, -0.01]**
Heterogeneity: Tau² = 0.00; Chi² = 1.71, df = 2 (P = 0.43); I² = 0%								
Test for overall effect: Z = 2.02 (P = 0.04)								
**1.4.2 Rheumatoid Arthritis**								
Adam et al. 2003	-0.1	2.9	30	0.9	1.7	30	26.4%	-0.42 [-0.93, 0.10]
Sköldstam et al. 2003	-3	3.8425	26	-0.4	3.8041	23	20.7%	-0.67 [-1.25, -0.09]
**Subtotal (95% CI)**			**56**			**53**	**47.1%**	**-0.53 [-0.91, -0.14]**
Heterogeneity: Tau² = 0.00; Chi² = 0.41, df = 1 (P = 0.52); I² = 0%								
Test for overall effect: Z = 2.69 (P = 0.007)								
**Total (95% CI)**			**122**			**109**	**100.0%**	**-0.45 [-0.71, -0.18]**
Heterogeneity: Tau² = 0.00; Chi² = 2.46, df = 4 (P = 0.65); I² = 0%								
Test for overall effect: Z = 3.32 (P = 0.0009)								
Test for subgroup differences: Chi² = 0.33, df = 1 (P = 0.57), I² = 0%								

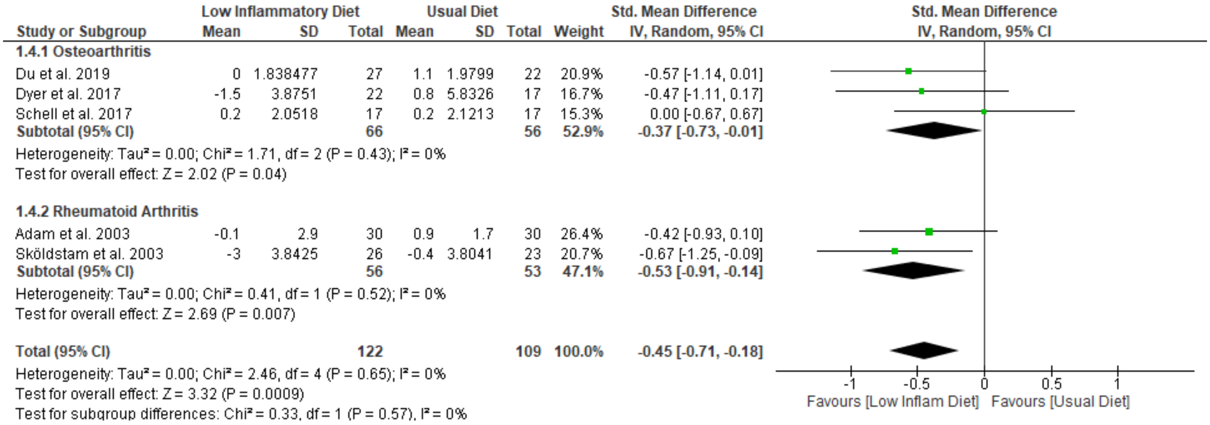

Supplementary Figure 2: Subgroup analysis of weight change by diagnosis following 2-4 months of intervention/control

Study or Subgroup	Low Inflammatory Diet Mean	SD	Total	Usual Diet Mean	SD	Total	Weight	Std. Mean Difference IV, Random, 95% CI
**1.5.1 Randomised Trials**								
Du et al. 2019	0	1.838477	27	1.1	1.9799	22	20.9%	-0.57 [-1.14, 0.01]
Dyer et al. 2017	-1.5	3.8751	22	0.8	5.8326	17	16.7%	-0.47 [-1.11, 0.17]
Schell et al. 2017	0.2	2.0518	17	0.2	2.1213	17	15.3%	0.00 [-0.67, 0.67]
Sköldstam et al. 2003	-3	3.8425	26	-0.4	3.8041	23	20.7%	-0.67 [-1.25, -0.09]
**Subtotal (95% CI)**			**92**			**79**	**73.6%**	**-0.46 [-0.76, -0.15]**
Heterogeneity: Tau² = 0.00; Chi² = 2.44, df = 3 (P = 0.49); I² = 0%								
Test for overall effect: Z = 2.92 (P = 0.004)								
**1.5.2 Prospective Study**								
Adam et al. 2003	-0.1	2.9	30	0.9	1.7	30	26.4%	-0.42 [-0.93, 0.10]
**Subtotal (95% CI)**			**30**			**30**	**26.4%**	**-0.42 [-0.93, 0.10]**
Heterogeneity: Not applicable								
Test for overall effect: Z = 1.59 (P = 0.11)								
**Total (95% CI)**			**122**			**109**	**100.0%**	**-0.45 [-0.71, -0.18]**
Heterogeneity: Tau² = 0.00; Chi² = 2.46, df = 4 (P = 0.65); I² = 0%								
Test for overall effect: Z = 3.32 (P = 0.0009)								
Test for subgroup differences: Chi² = 0.02, df = 1 (P = 0.89), I² = 0%								

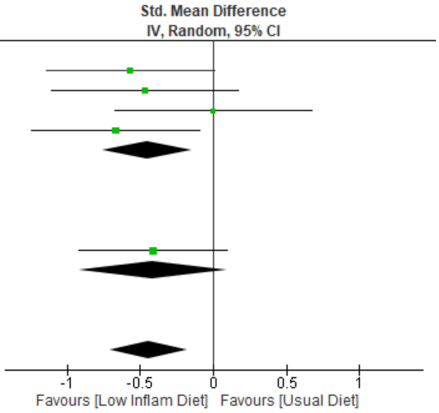

Supplementary Figure 3: Subgroup analysis of weight change by study type following 2-4 months of intervention/control

A) Change in CRP at 2-4 months

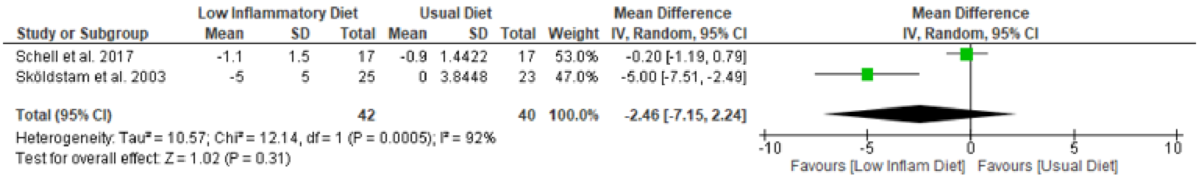

B) Change in IL-6 at 2-4 months

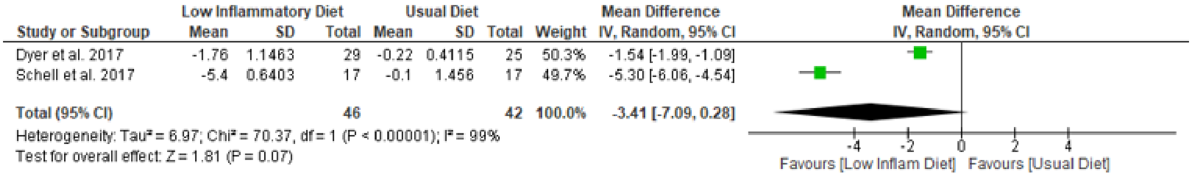

C) Change in IL-1β at 2-4 months

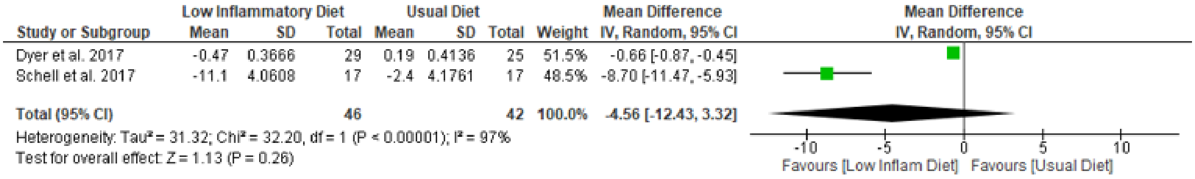

Supplementary Figure 4: Meta-analysis of change in specific inflammatory biomarkers after 2-4 months of intervention/control.

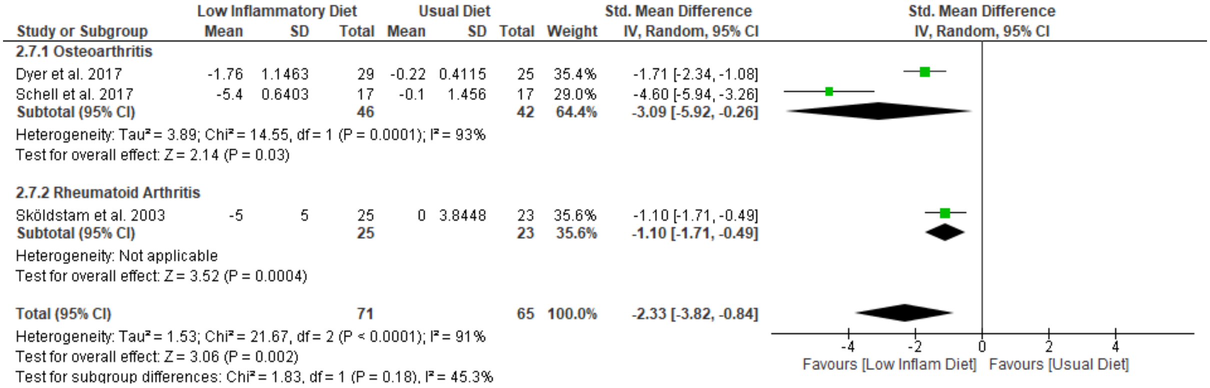

Supplementary Figure 5: Meta-analysis of change in any inflammatory biomarker by diagnosis after 2-4 months of intervention/control.

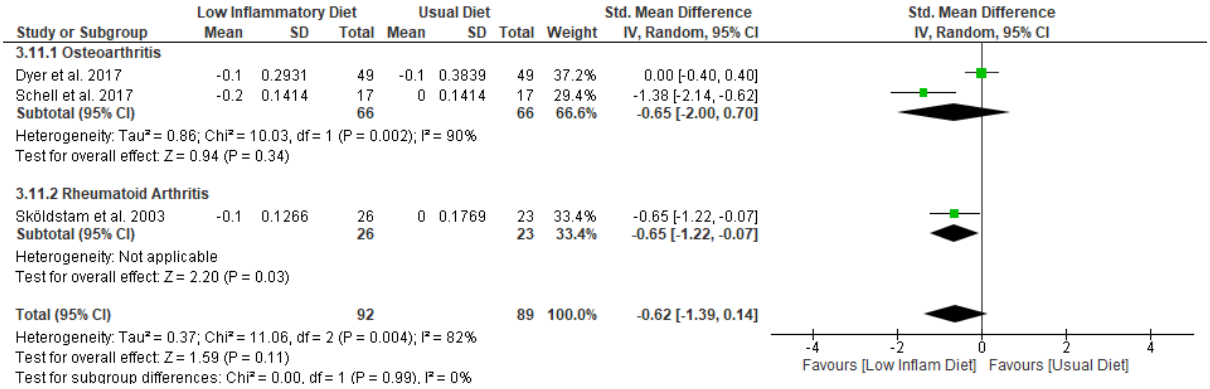

Supplementary Figure 6: Meta-analysis of change in any physical function outcomes by diagnosis after 2-4 months of intervention/control.

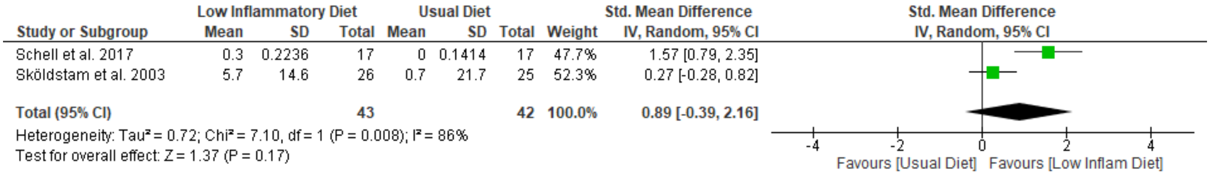

Supplementary Figure 7: Meta-analysis of change in any general health measure after 2-4 months of intervention/control.

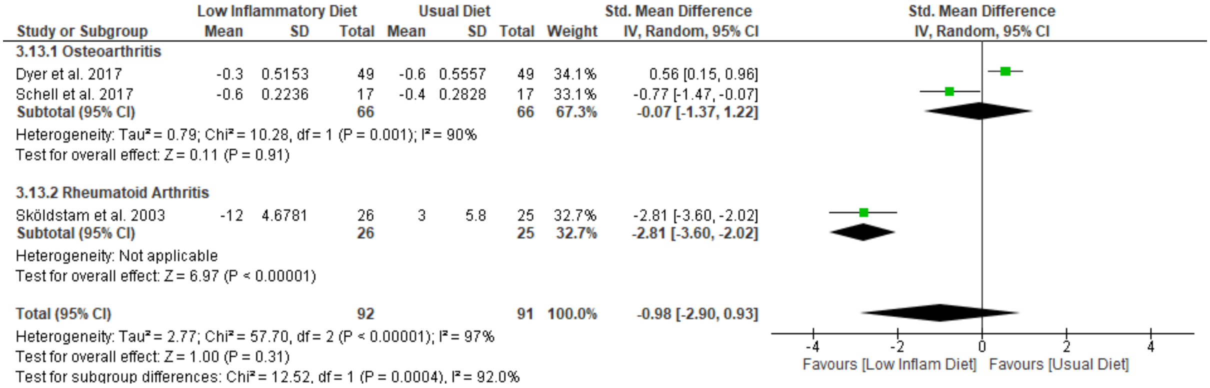

Supplementary Figure 8: Meta-analysis of change in any pain scores by diagnosis after 2-4 months of intervention/control.

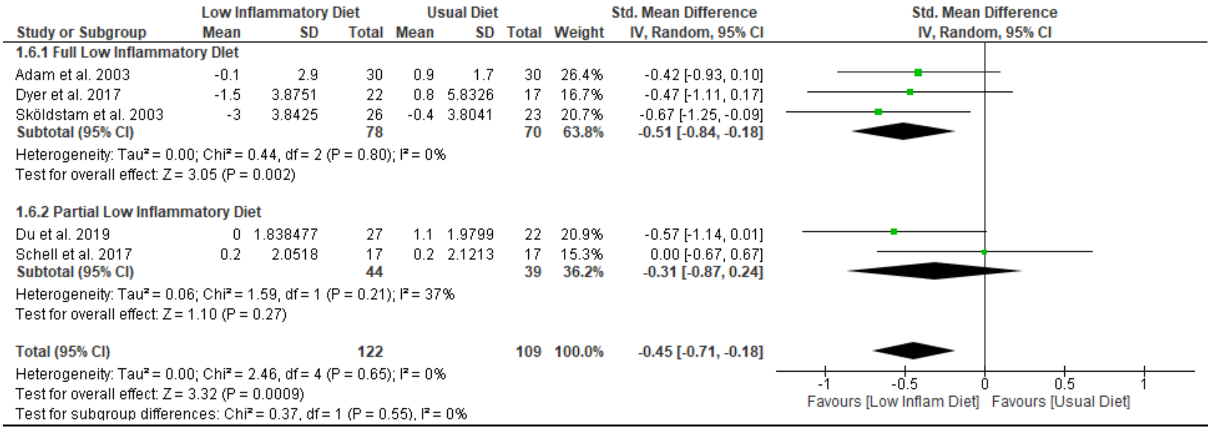

Supplementary Figure 9: Subgroup analysis of weight change by nature of intervention (full vs partial low-inflammatory diet) following 2-4 months of intervention/control.

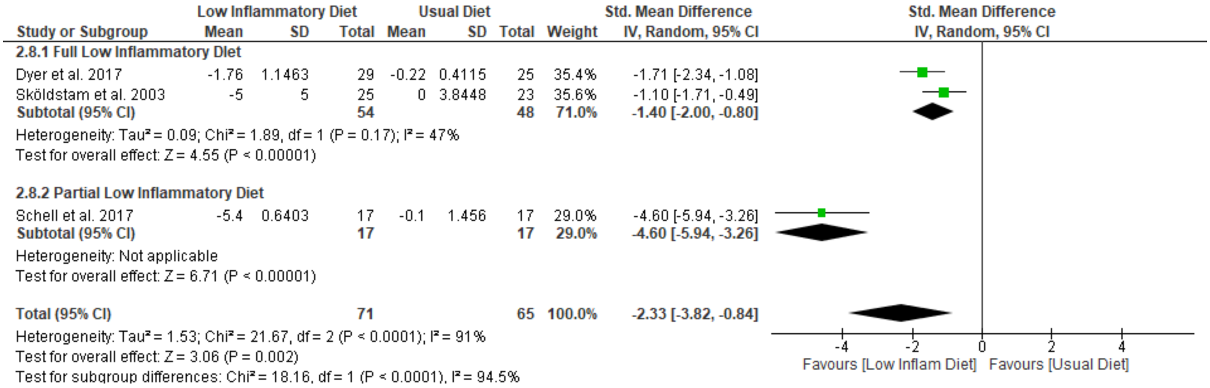

Supplementary Figure 10: Meta-analysis of change in any inflammatory biomarker nature of intervention (full vs partial low-inflammatory diet) after 2-4 months of intervention/control (Note: A meta-analysis comparing interventions that resulted in weight loss compared to no weight loss produced the same analysis. Weight loss included the same trials as full low-inflammatory diet, no weight loss included the same trials as partial low-inflammatory diet).

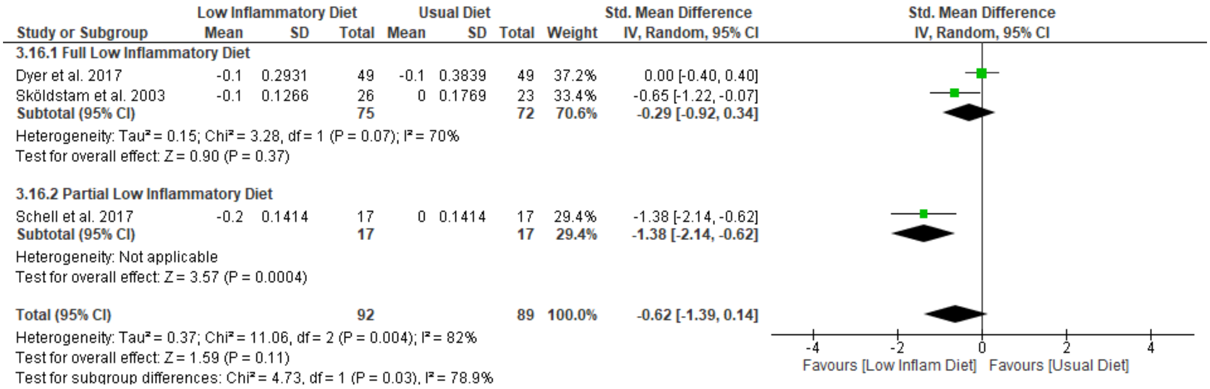

Supplementary Figure 11: Meta-analysis of change in any physical function outcomes by nature of intervention (full vs partial low-inflammatory diet) after 2-4 months of intervention/control. (Note: A meta-analysis comparing interventions that resulted in weight loss compared to no weight loss produced the same analysis. Weight loss included the same trials as full low-inflammatory diet, no weight loss included the same trials as partial low-inflammatory diet).

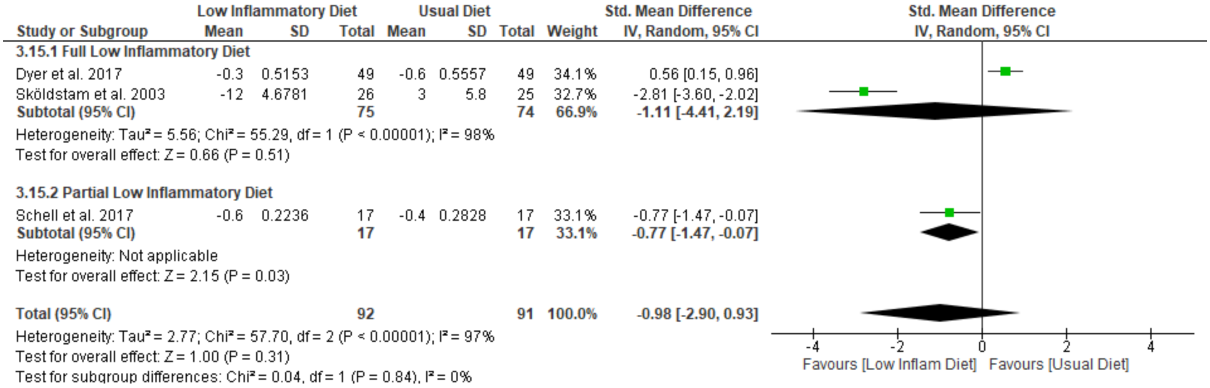

Supplementary Figure 12: Meta-analysis of change in any pain scores by nature of intervention (full vs partial low-inflammatory diet) after 2-4 months of intervention/control. (Note: A meta-analysis comparing interventions that resulted in weight loss compared to no weight loss produced the same analysis. Weight loss included the same trials as full low-inflammatory diet, no weight loss included the same trials as partial low-inflammatory diet).

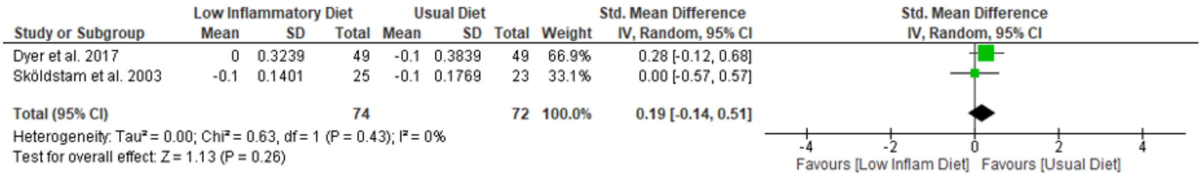

__Supplementary Figure 13: Meta-analysis of physical function change when comparing low inflammatory diet to usual diet following 0-2 months of the intervention/control.__

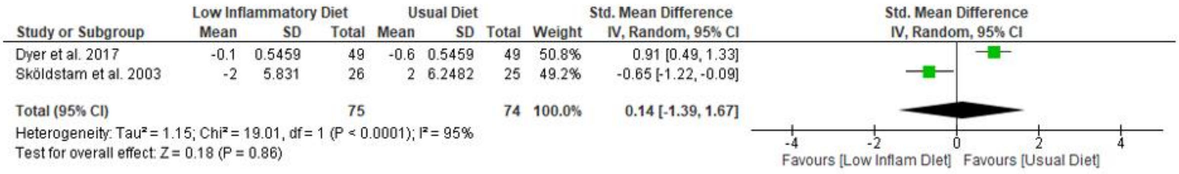

__Supplementary Figure 14: Meta-analysis of pain score change when comparing low inflammatory diet to usual diet following 0-2 months of the intervention/control.__

SEARCH SYNTAX:

A. Syntax for MEDLINE, EMBASE, CENTRAL and Cochrane Databases for Systematic Reviews (note: this syntax would be used using the OvidSP Platform searching through the four databases):

1. arthritis/
2. arthritis, psoriatic/
3. exp arthritis, rheumatoid/
4. exp osteoarthritis/
5. (arthritis adj5 (psoriatic or psoriasis)).tw.
6. (arthritis adj5 rheumatoid).tw.
7. (arthritis adj5 (seronegative or seropositive)).tw.
8. osteoarthritis.tw.
9. arthritis, reactive/
10. spondylitis, ankylosing/
11. (arthritis adj5 reactive).tw.
12. (spondylitis adj5 ankylosing).tw.
13. (arthritis adj5 IBD-related).tw.
14. (arthritis adj5 IBD-associated).tw.
15. or/1-14
16. limit 15 to "all adult (19 plus years)"
17. (low Inflammatory adj3 diet*).tw.
18. (low Inflammatory adj3 dietary pattern*).tw.
19. (low Inflammatory adj3 type diet*).tw.
20. (anti-Inflammatory adj3 diet*).tw.
21. (anti-Inflammatory adj3 dietary pattern*).tw.
22. (anti-Inflammatory adj3 type diet*).tw.
23. Diet, Mediterranean/
24. (mediterranean adj3 diet*).tw.
25. (mediterranean adj3 dietary pattern*).tw.
26. (mediterranean adj3 type diet*).tw.
27. MedDiet.tw.
28. MeDi.tw.
29. MeDiet.tw.
30. ((crete or cretan) adj4 diet*).tw.
31. animals/ not (animals/ and humans/)
32. or/17-30
33. 16 and 32
34. 33 not 31
35. remove duplicates from 34

B. Syntax for CINAHL

S1	arthritis
S2	psoriatic arthritis
S3	rheumatoid arthritis
S4	osteoarthritis
S5	arthritis N5 (psoriasis OR psoriatic)
S6	arthritis N5 (rheumatoid)
S7	arthritis N5 (seronegative OR seropositive)
S8	TX osteoarthritis
S9	reactive arthritis
S10	ankylosing spondylitis
S11	arthritis N5 (reactive)
S12	spondylitis N5 (ankylosing)
S13	arthritis N5 (IBD-related)
S14	arthritis N5 (IBD-associated)
S15	S1 OR S2 OR S3 OR S4 OR S5 OR S6 OR S7 OR S8 OR S9 OR S10 OR S11 OR S12 OR S13 OR S14
S16	S1 OR S2 OR S3 OR S4 OR S5 OR S6 OR S7 OR S8 OR S9 OR S10 OR S11 OR S12 OR S13 OR S14 Limiters - Human; Age Groups: Adult: 19-44 years, Middle Aged: 45-64 years, Aged: 65+ years, Aged, 80 and over
S17	low inflammatory N3 (diet)
S18	low inflammatory N3 (dietary pattern)
S19	low inflammatory N3 (type diet)
S20	mediterranean diet
S21	Mediterranean N3 (Diet)
S22	Mediterranean N3 (Dietary pattern)
S23	Mediterranean N3 (type diet)
S24	TX MedDiet
S25	TX MeDi
S26	TX MeDiet
S27	(crete or cretan) N4 diet
S28	Anti-inflammatory N3 (diet)
S29	Anti-inflammatory N3 (dietary pattern)
S30	anti-inflammatory N3 (type diet)
S31	S17 OR S18 OR S19 OR S20 OR S21 OR S22 OR S23 OR S24 OR S25 OR S26 OR S27 OR S28 OR S29 OR S30
S32	S17 OR S18 OR S19 OR S20 OR S21 OR S22 OR S23 OR S24 OR S25 OR S26 OR S27 OR S28 OR S29 OR S30 Limiters - Human
S33	S16 AND S32
